# Supplementary material for: Unveiling a flip-over retention mechanism in the gas-phase Cl− + (CH3)3CI SN2 reaction
Source: Nat Commun. 2026 May 1;17:3947. doi: 10.1038/s41467-026-72121-4 (PMC13134954; doi:10.1038/s41467-026-72121-4)
Supplement: Supplementary file 1 — Supplementary Information [file 41467_2026_72121_MOESM1_ESM.pdf]

Supplementary information:

Unveiling a flip-over retention mechanism in the gas-phase $Cl^- + (CH_3)_3CI$ S_N2 reaction

Xiaoxiao Lu,[1,2] Jennifer Meyer[3,4], Lulu Li,[5] Eduardo Carrascosa,[3] Björn Bastian,[3] Tim Michaelsen,[3] Bina Fu,[2,6,7*], Dong H. Zhang[2,6,7], Roland Wester[3*]

[1] *Interdisciplinary Research Center for Biology and Chemistry, Liaoning Normal University, Dalian, 116029, China*

[2] *State Key Laboratory of Chemical Reaction Dynamics, Dalian Institute of Chemical Physics, Chinese Academy of Sciences, Dalian 116023, China*

[3] *Institut für Ionenphysik und Angewandte Physik, Universität Innsbruck, Technikerstraße 25, 6020 Innsbruck, Austria*

[4] *Fachbereich Chemie und Forschungszentrum OPTIMAS, RPTU Kaiserslautern-Landau, Erwin-Schrödinger Straße 52, 67663 Kaiserslautern, Germany*

[5] *National Engineering Research Center of Lower-Carbon Catalysis Technology, Dalian Institute of Chemical Physics, Chinese Academy of Sciences, Dalian 116023, China*

[6] *University of Chinese Academy of Sciences, Beijing 100049, China*

[7] *Hefei National Laboratory, Hefei 230088, China*

Supplementary Notes

Supplementary Note 1

In this study, we developed a new, accurate, full-dimensional PES of the Cl^- + $(CH_3)_3CI$ gas-phase reaction, involving both the base-induced elimination (E2) and the bimolecular nucleophilic substitution (S_N2) processes. This PES was constructed based on a total of roughly 256,000 energy points, computed using CAM-XYG3/aug-cc-pVTZ(aug-cc-pVTZ-PP for iodine atom)[1,2] level of theory, and employing the fundamental invariant neural network (FI-NN) [3,4] fitting method.

Given the significant complexity of electronic structure calculations for the title reaction system, which consists of 15 atoms and 104 electrons, we selected the recently proposed hybrid functional CAM-XYG3 developed by Xu and coworkers[1,2]. CAM-XYG3 represents a hybrid approach that combines the favorable characteristics of XYG3[2] with a long-range correction utilizing the Coulomb-attenuating method

(CAM)[1]. Our previous work[5] has demonstrated that the CAM-XYG3 method achieves high accuracy levels comparable to the benchmark explicitly correlated coupled-cluster (CCSD(T)) method. In the present investigation, we calculated the energies of all collected geometries employing the hybrid function CAM-XYG3 implemented within the Gaussian 16 (Revision A.03) software package[6], together with Dunning's augmented correlation-consistent triple-zeta (aug-cc-pVTZ) basis set and the corresponding aug-cc-pVTZ-PP basis set for the iodine atom. It takes about 40 minutes of real CPU time for a single point using five threads on one computer node.

Following our prior work for the $F^- + (CH_3)_3CI$ reaction, we adopted the same strategy, that is, space partitioning and energy splitting methods to effectively address the challenge associated with fitting our extensive dataset. Comprehensive details can be found in the supplementary section of this document[5]. In summary, we divided the configuration space into three distinct domains, and the total energy of the geometries at the entrance of the reaction or the exit of the E2 channel was separated into combined components of monomer energy and corresponding interaction energy. In addition, the flexible FI-NN method was employed for PES fitting, which can minimize the number of terms compared to the permutationally invariant polynomial (PIP)[7,8] method. During the course of neural network training, we employed the Levenberg-Marquardt algorithm[9] to iteratively update the weights and biases, aiming to achieve an optimal fit. The root mean square error (RMSE), as defined in Equation (1), was applied to measure the fitting accuracy.

$$\mathrm{RMSE}=\sqrt{\frac{1}{n}\sum_{i=1}^{n}(E_{\mathrm{fit}}-E_{abinitio})^2} \qquad (1)$$

It is important to note that the size of the neural network structure can significantly impact the accuracy of the fitting process. Consequently, a meticulous series of fitting tests was conducted for each region to determine the optimal parameters. For easy reference, Supplementary Table 1 includes the construction details of the current PES, the space partitions, neural network structures, the number of data points, and their corresponding RMSE.

Supplementary TABLE 1: Number of energy points, NN structure, and fitting errors (in meV) of different parts of the FI-NN PES

	Partition	*Ab initio*	NN structure	RMSE(meV)
**Part 1**				
**Cl^- +$(CH_3)_3CI$[a]**	$R_{C\text{-}Cl}$ > 5.5Å	51168	500-3-30-1	0.94
**$(CH_3)_3CI$**		72362	500-10-50-1	9.0
**Part 2**				
Interaction region	Except Part 1 and Part 3	181521	500-70-100-1	21.3
**Part 3**				
**$(CH_3)_2CCH_2$ + I^- + HCl[a]**	$R_{C\text{-}Cl/C\text{-}I/Cl\text{-}I}$ > 5.0Å	23872	500-10-20-1	3.3
**$(CH_3)_2CCH_2$**		35845	606-10-100-1	4.8
**HCl**		240	1-5-10-1	0.002
**Total**				18.3

[a] interaction energy surface

In the process of configuration collection, we initially employed direct dynamics simulations based on the unrestricted B3LYP/6-31+G* level of theory to generate the initial dataset, starting from the initially guessed stationary points along the S_N2 and E2 pathways. Additional geometries were gradually added into the dataset through further QCT calculations based on the preliminary PES and the updated PESs. Data points that were either too close either in the energy domain or geometry domain were discarded to enhance the efficiency of the FI-NN fitting. Furthermore, configurations along the minimum energy path, as well as all optimized stationary points were added to the corresponding dataset.

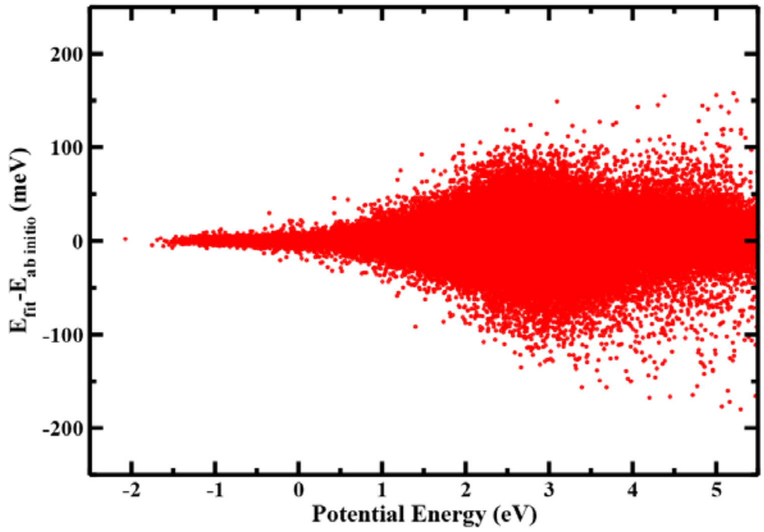

Supplementary Figure 1 | Fitting quality. Fitting errors for all data points on the FI-NN PES as a function of their corresponding CAM-XYG3/AVTZ(-PP) energies relative to the energy of Cl^- + $(CH_3)_3CI$.

Overall, the total RMSE for all data points in the three parts amounts to 18.3 meV (0.42 kcal mol^{-1}). Figure S1 shows the fitting errors associated with each data point in relation to their corresponding CAM-XYG3/AVTZ(-PP) energies. Despite the wide energy range of the current PES (~ 8 eV), the *ab initio* energies are well reproduced by the FI-NN fitting. In Fig. S2, we depict all stationary points along the reaction pathways, with critical bond lengths indicated. It is essential to highlight the significant computational time required for direct optimization using the CAMXYG3/AVTZ(-PP) method, due to the necessity of calculating numerical forces in the absence of analytical force expressions for this method. As an alternative approach, we used the CCSD method implemented by Molpro 2023.1 program package[10] together with the AVDZ(-PP) basis for optimization, except for the BS-TS, for which the B3LYP/6-311G** method was employed. We found that the resulting geometries are in good agreement with those on the PES. The energies obtained from the potential energy surface were compared with those calculated by the CAM-XYG3/AVTZ(-PP) method, showing good agreement.

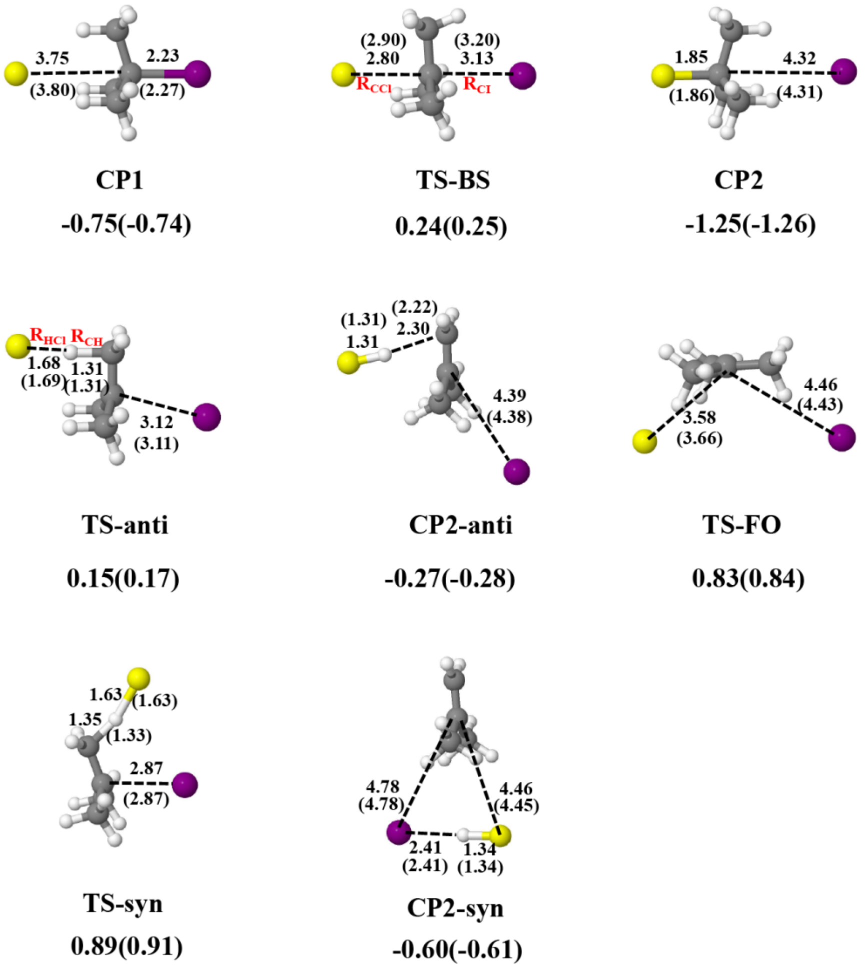

Supplementary Figure 2 | Optimized configurations. Optimized geometries of the stationary points along the E2 and S_N2 reaction pathways on the FI-NN PES, along with benchmark CCSD/AVDZ(-PP) calculations (in brackets) except for the BS-TS, for which the B3LYP/6-311G** method was employed, with key bond lengths indicated (in Å). The corresponding energies were obtained from the FI-NN PES and the CAM-XYG3/AVTZ(-PP) method (in brackets).

In order to further verify the accuracy of the full-dimensional potential energy surface, we present contour plots for the two principal reaction channels: the back-side attack S_N2 and *anti*-E2 reactions, as illustrated in Fig. S3. These contour plots are constructed as functions of the relevant bond formation and cleavage processes (specifically $R_{C\alpha\text{-}Cl}$ and $R_{C\alpha\text{-}I}$ for S_N2; $R_{H\beta\text{-}Cl}$ and $R_{C\beta\text{-}H\beta}$ for E2) while optimizing all vibrational degrees of freedom on the current PES. Fig. S3 presents the prominent features of the potential energy hypersurface, showcasing the shared ion-dipole complex (CP1) between back-side S_N2 and *anti*-E2, the reaction energy barriers, as well as the post-reaction complexes, with their positions and energies accurately described.

Overall, the PES exhibits remarkable smoothness over the entire configuration space. The contour plots further demonstrate the high accuracy of the fitting and the reliability of 15-atom multi-channel PES, as well as the robust capability of the FI-NN methodology.

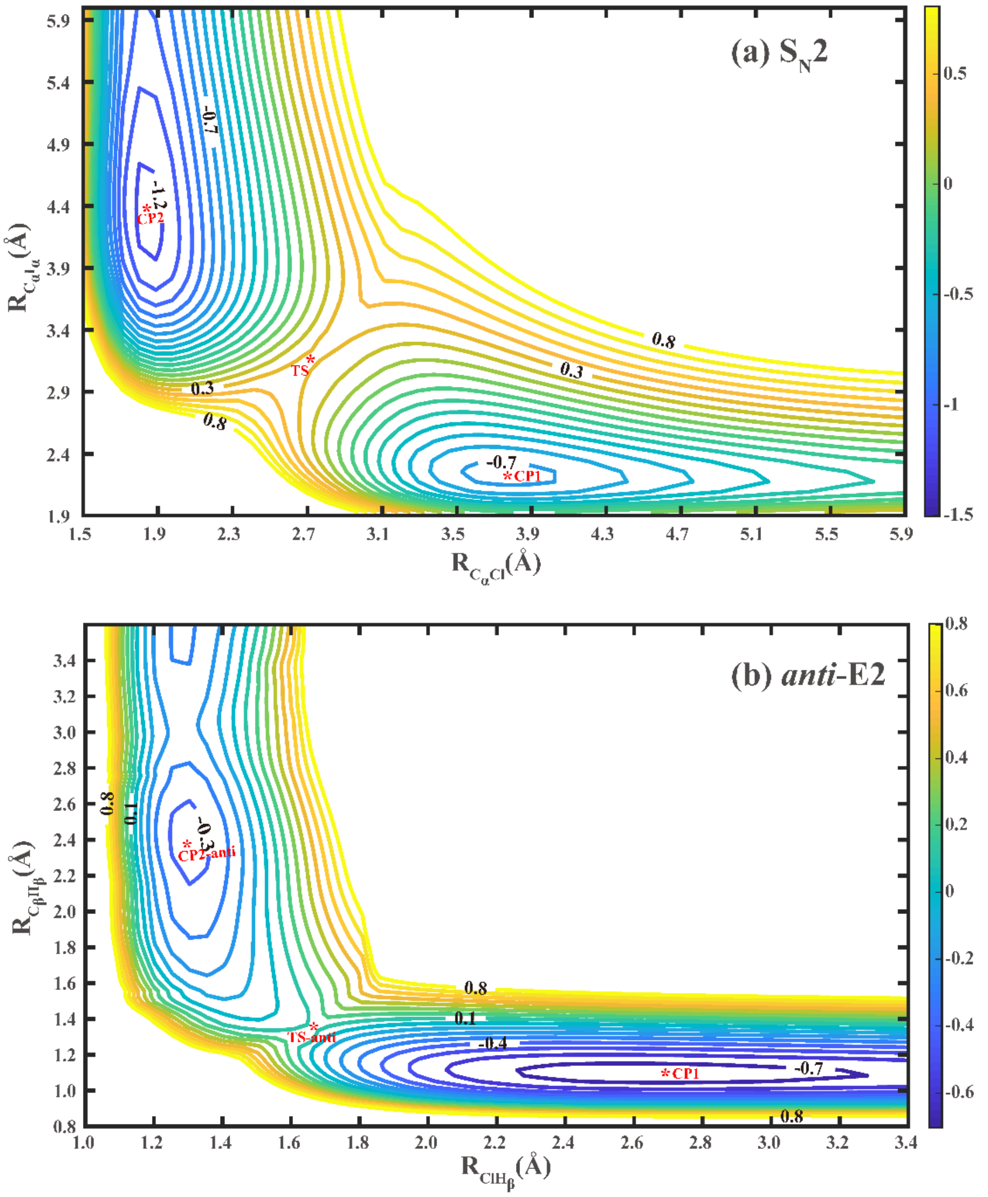

Supplementary Figure 3 | Contour plots. Contour plots illustrating the back-side attack S_N2 **(a)** and *anti*-E2 **(b)** channels on the FI-NN PES obtained from relaxed scans along the two reactive bond lengths, with all other degrees of freedom fully optimized.

Supplementary Note 2

To investigate the dynamics of the Cl^- + $(CH_3)_3CI$ reaction, we conducted standard

QCT calculations[11,12] at collision energies of 1.1 eV and 1.9 eV, with $(CH_3)_3CI$ initially in its ground rovibrational state. Initial coordinates and momenta of $(CH_3)_3CI$ were acquired through random sampling of normal coordinates and momenta, followed by subsequent adjustments to enforce zero angular momentum. The initial separation between the reactants' centers of mass was computed as $(x^2 + b^2)^{1/2}$, with x set to 38.0 bohr due to the large long-range interactions in the entrance channel. The impact parameter b was scanned from 0 to b_{max} (10.0 bohr for 1.1 eV and 9.0 bohr for 1.9 eV) with an interval of 0.5 bohr. Trajectories were carried out using the Velocity-Verlet integration algorithm with a time step of 0.024 fs, terminating when fragments separated by 30 bohr or returned to reactants. For the QCT calculations, analytical gradients were employed, significantly enhancing computational speed by approximately tenfold compared to using numerical gradients. A total of roughly 4.2 million and 4.7 million trajectories were run at the two high collision energies.

We statistically analyzed the inversion and retention S_N2 cases by examining the changes in the dihedral angles formed by the three β-C atoms and the central carbon atom. Through analysis, we observed that some trajectories of the E2 reaction violated from the zero-point energy of the product owing to the endothermic nature of the E2 pathway. Thus, the QCT analysis of the E2 reaction takes into account trajectories that include a soft ZPE constraint, where the total vibrational energies of the HCl and $(CH_3)_2CCH_2$ products are not lower than the sum of their respective zero-point energy. In contrast, no ZPE violation was found to the products of S_N2 reaction events, due to the large exothermicity of this channel. As a result, we collected all S_N2 trajectories for analysis.

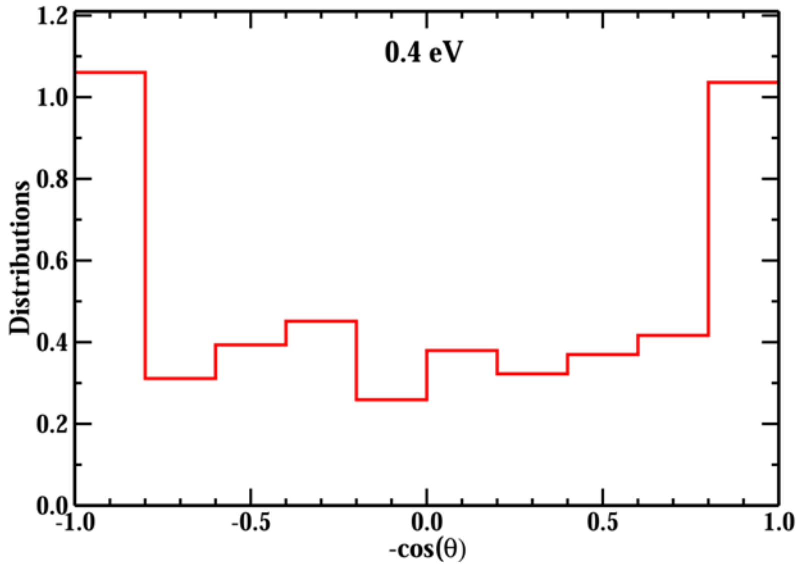

Supplementary Figure 4 | Theoretical angular distribution. Normalized angular distribution of product I^- contributed by all E2 and S_N2 channels at the collision energy of 0.4 eV, showing a forward-backward symmetric pattern.

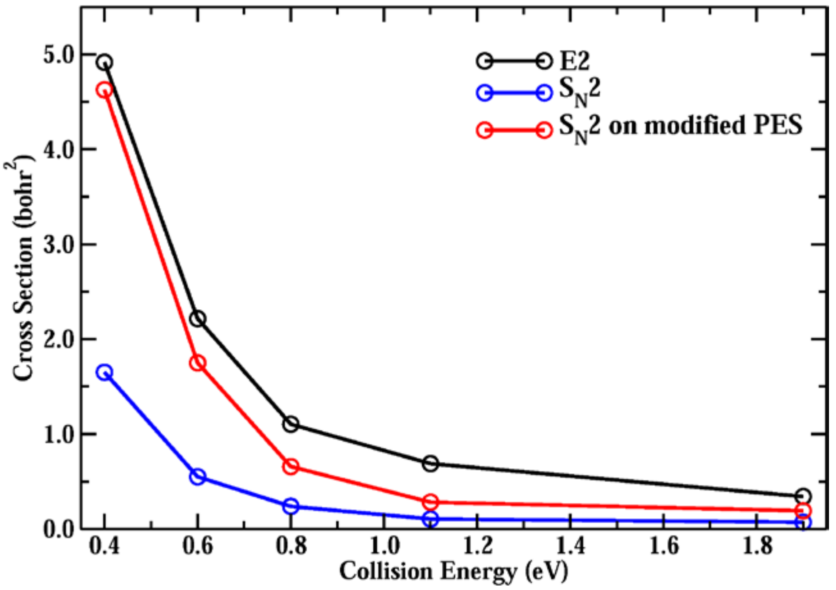

Supplementary Figure 5 | Integral cross sections (ICS). Cross sections as a function of collision energy for the E2 and S_N2 reactions, and for the S_N2 reaction on

the modified PES. The analysis for the E2 products is performed using the softZPE constraint.

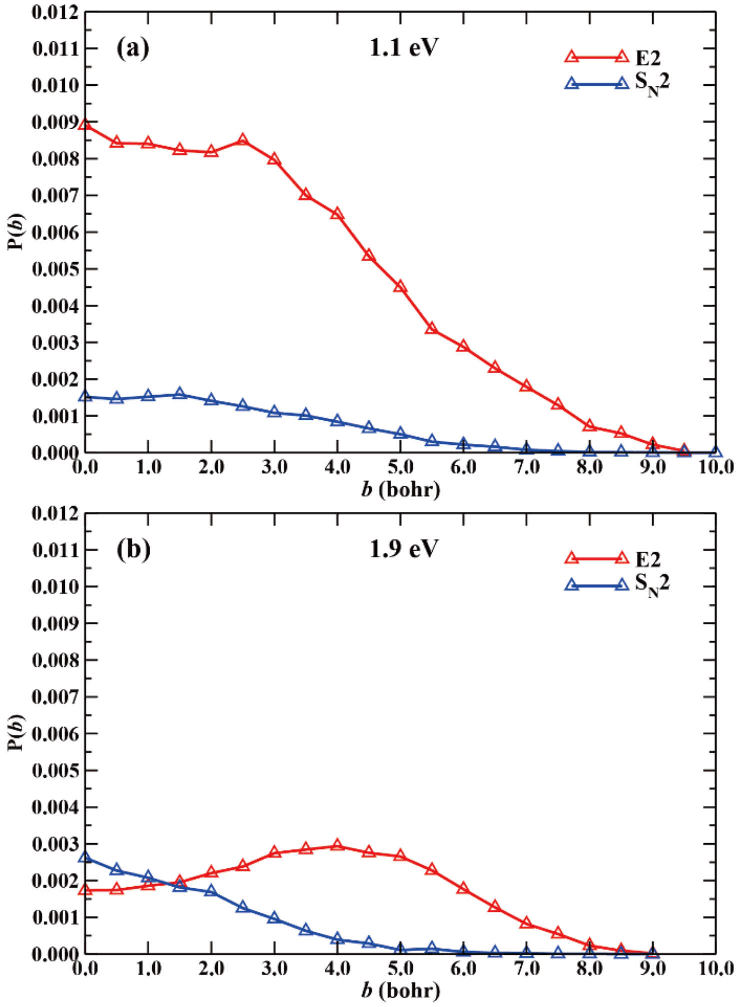

Supplementary Figure 6 | Opacity functions. Reaction probabilities of the E2 (in red) and S_N2 (in blue) mechanisms as a function of impact parameter at collision energies of 1.1 eV **(a)** and 1.9 eV **(b)** obtained from QCT calculations.

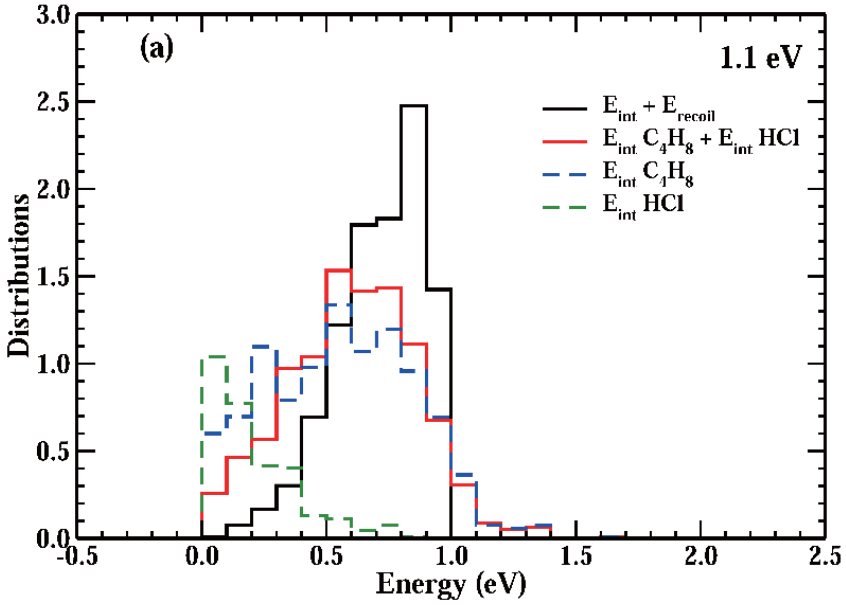

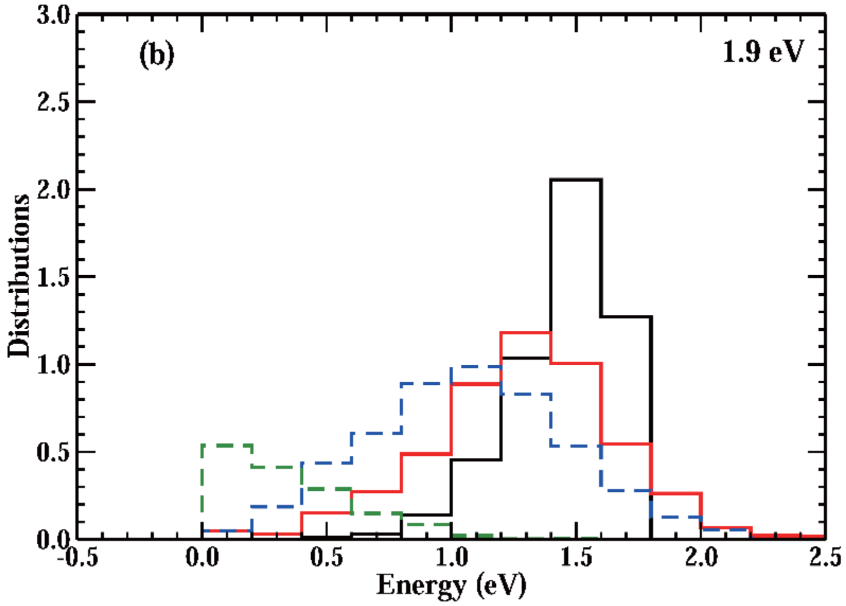

Supplementary Figure 7 | Internal energy distributions. a-b, Internal energy distributions (in black) at collision energies of 1.1 eV **(a)** and 1.9 eV **(b)** obtained from the QCT simulations. The dashed lines represent the individual internal energy distributions of the products, $(CH_3)_2CCH_2$ (in blue) and HCl (in green), with the sum of both distributions shown by the solid red lines. The difference between the red and black distributions indicates the energy channeled into the recoil between the two neutral products. For clarity, the red, blue, and green lines have been normalized to 0.5, 0.5, and 0.2, respectively.

Supplementary References

1. Yanai, T., Tew, D. P. & Handy, N. C. A new hybrid exchange–correlation functional using the Coulomb-attenuating method (CAM-B3LYP). *Chem. Phys. Lett.* **393,** 51-57 (2004).
2. Zhang, Y., Xu, X. & Goddard, W. A. Doubly hybrid density functional for accurate descriptions of nonbond interactions, thermochemistry, and thermochemical kinetics. *Proc. Natl. Acad. Sci. U.S.A.* **106,** 4963-4968 (2009).
3. Shao, K., Chen, J., Zhao, Z. & Zhang, D. H. Communication: Fitting potential energy surfaces with fundamental invariant neural network. *J. Chem. Phys.* **145,** 071101 (2016).
4. Chen, R., Shao, K., Fu, B. & Zhang, D. H. Fitting potential energy surfaces with fundamental invariant neural network. II. Generating fundamental invariants for molecular systems with up to ten atoms. *J. Chem. Phys.* **152,** 204307 (2020).
5. Lu, X. et al. Unexpected steric hindrance failure in the gas phase F^{-} + $(CH_3)_3CI$ S_N2 reaction. *Nat. Commun.* **13,** 4427 (2022).
6. Frisch, M. J. et al. Gaussian 16 Rev. A.03. Wallingford, CT; 2016.
7. Braams, B. J. & Bowman, J. M. Permutationally invariant potential energy surfaces in high dimensionality. *Int. Rev. Phys. Chem.* **28,** 577-606 (2009).
8. Bowman, J. M., Czakó, G. & Fu, B. High-dimensional ab initio potential energy surfaces for reaction dynamics calculations. *Phys. Chem. Chem. Phys.* **13,** 8094-8111 (2011).
9. Hagan, M. T. & Menhaj, M. B. Training feedforward networks with the Marquardt algorithm. *IEEE Trans. Neural Networks* **5,** 989-993 (1994).
10. Werner, H. J., Knowles, P. J., Knizia, G., Manby, F. R. & Schtz, M. Molpro: a general-purpose quantum chemistry program package. *WIREs Comput Mol Sci* **2,** 242--253 (2012).
11. Hase, W. L. Classical Trajectory Simulations: Initial Conditions. In: von Ragué Schleyer NLA, T. Clark, J. Gasteiger, P. A. Kollman, H. F. Schaefer and P. R. Schreiner (ed). *Encyclopedia of Computational Chemistry*, vol. 1. Wiley: New York, 2002, pp 402–407.
12. Hase, W. L. Classical Trajectory Simulations: Final Conditions. In: von Ragué Schleyer NLA, T. Clark, J. Gasteiger, P. A. Kollman, H. F. Schaefer and P. R. Schreiner (ed). *Encyclopedia of Computational Chemistry*, vol. 1. Wiley: New York, 2002, pp 399-402.
