# Supplementary material for: Unveiling a flip-over retention mechanism in the gas-phase Cl− + (CH3)3CI SN2 reaction
Source: Nat Commun. 2026 May 1;17:3947. doi: 10.1038/s41467-026-72121-4 (PMC13134954; doi:10.1038/s41467-026-72121-4)
Supplement: Supplementary file 2 — Description of Additional Supplementary File [file 41467_2026_72121_MOESM2_ESM.pdf]

The Description of Additional Supplementary Files

Supplementary Movie 1: An animation for flip-over mechanism with forward scattering

Supplementary Movie 2: An animation for flip-over mechanism with backward scattering
